# The prevalence of ocular diseases in polish Arabian horses

**DOI:** 10.1186/s12917-017-1252-8

**Published:** 2017-11-07

**Authors:** Katarzyna Paschalis-Trela, Anna Cywińska, Jan Trela, Michał Czopowicz, Jerzy Kita, Lucjan Witkowski

**Affiliations:** 1 Laboratory of Veterinary Epidemiology and Economics, Faculty of Veterinary Medicine, Warsaw University of Life Sciences-SGGW, Warsaw, Nowoursynowska 159c, 02-786 Warsaw, Poland; 2 TRELA VETs Referrals, Warsaw, Poland; 3 Division of Pathophysiology, Department of Pathology and Veterinary Diagnostics, Faculty of Veterinary Medicine, Warsaw University of Life Sciences-SGGW, Nowoursynowska 159c, 02-786 Warsaw, Poland

**Keywords:** Epidemiology, Equine, Eye, ERU, Horse, Uveitis

## Abstract

**Background:**

Equine ocular diseases pose a medical challenge due to long-lasting and cost-consuming therapies as well as economic issues associated with potential decrease in value of affected horses. The scale of the problem is significant but difficult to precisely define because epidemiological data is limited and lacks consistency in presentation. To date, no retrospective studies specifically investigating Arabian horses have been published.

**Results:**

The aim of this cross-sectional study was to investigate the prevalence of ocular lesions and define the ocular diseases present in Arabian horses from breeding farms in Poland. Clinical and ophthalmic examination of 615 Arabian horses at Polish breeding farms (15% of Arabian population in Poland) were performed and medical history from the previous 5 years was analyzed. Data was obtained from review of veterinary archives and epidemiological interview of the resident veterinarian at each farm. The prevalence of ocular diseases was 9.75%. The following pathologies were diagnosed (with their respective prevalence): equine recurrent uveitis (ERU; 5.5%); cataract not related to ERU (3.3%); non-visual eyes (1.13%); posttraumatic lesions (0.8%); glaucoma (0.16%).

**Conclusions:**

In this study, ERU was the most common ocular disease identified in Arabian horses in Poland. Its prevalence was lower than usually reported in Europe and the United States. There was no sex or farm predisposition but ocular disease prevalence increased with age. Other severe ocular pathologies were also observed, confirming that ocular diseases remain an important clinical problem.

**Electronic supplementary material:**

The online version of this article (10.1186/s12917-017-1252-8) contains supplementary material, which is available to authorized users.

## Background

The Polish Arabian horse breeding program is considered one of the largest and the most successful worldwide, as evidenced by the considerable material value and global interest in the horses being sold. For example, in 2015 at the high-profile Pride of Poland Arabian Horse Auction the mare Pepita sold for the high dollar amount of €1.4 million ($US 1.6 m; $NZ 2.5 m). Three hundred years of tradition and many years of international success have contributed to the importance of Polish horses for the Arabian and Warmblood breeds [1, 2].

It is widely accepted that ocular diseases and related complications are a serious health problem worldwide with great impact on quality of life and value of affected horses, as well as athletic and show potential or use for other purposes. In addition to the pain associated with these conditions that can impair welfare, eye disorders may lead to partial or full blindness, which may exclude the horse from performance occupations and cause potential risk for the rider. Ocular diseases often require long-lasting and expensive treatments, and particularly if vision-threatening, may lead to disruption of training and potential disqualification from competition due to ongoing therapies and impairment. Thus, these diseases lead to large financial losses in the equine industry, estimated at millions of dollars per year in the US [3].

It is difficult to define the scale of the problem and associated occurrence of ocular diseases in Poland where equine ophthalmology is a relatively new veterinary discipline and epidemiological data is lacking. To date, no reports have been published on the prevalence and type of ocular lesions in Arabian horse breeding farms. A limited number of epidemiological studies have been published but none have focused on a large group of horses of one specific breed [3–12].

Thus, the aim of this study was to determine the prevalence of ocular pathologies in the Arabian horse population of Poland.

## Methods

This study was approved by the 3rd Local Commission for Ethics in Animal Experiments (Decision No. 24/2011) and carried out in 2011–2013. All horses included in this cross-sectional study were recorded in the Polish Arabian Stud Book and their pedigree confirmed with an official passport. At the time the study was designed, the Arabian horse population in Poland numbered approx. 4000 individuals, however most of them were kept in several hundred small stables. There were only three large breeding farms constituting 615 Arabian horses total (roughly 15% of the general population) – 292, 270 and 53 animals from each farm, respectively. A minimum required sample size to estimate prevalence of ocular diseases at expected true prevalence of 50%, precision of 5% and 95% level of confidence was determined to be 352 individuals using EpiTools [13]. As 615 horses were available, they all were enrolled in the study. Sex and age were recorded for each animal and they were assigned to three age/use classes per E.C.A.H.O (European Conference of Arabian Horse Organization) recommendations: junior (up to 5 years), main reproduction age (5–12 years) and older horses (over 12 years).

Each horse underwent general clinical and ophthalmological examination of both eyes according to commonly accepted standards [3, 5, 14–16]. Medical histories from the preceding five years were reviewed. Clinical reports provided by each staff veterinarian were utilized to determine relevant medical details such as the recurrence of uveitic episodes. Ophthalmological examination was performed in horses without sedation or nerve blocks, and all examinations were conducted by a single investigator in a dark stable or in the predawn morning to ensure proper dark adaptation. Both eyes were examined with a penlight and slit-lamp (Kowa SL15). Intraocular pressure was measured (Tono-Pen XL, Mentor, USA) after use of topical anesthetic (Alcaine® proparacaine hydrochloride ophthalmic solution, USP 0.5%). Both pupils were dilated with Tropicamide 1% (Polfa, Poland) followed by a direct ophthalmoscopy (Heine EN 20-1d). When indicated (e.g. partially opaque cornea), ocular ultrasonography was carried out to evaluate the state of the globe and internal structures (Portable Ultrasound Machine GE LOGIQ I 10 MHz). Horses with suspected differences in globe size underwent ultrasonography as well. The measurement results allowed evaluation for conditions such as buphthalmos. Glaucoma diagnosis was made based on both case history and clinical signs, including corneal edema with endothelial striae, elevated IOP >30 mmHg, and globe enlargement.

Normality of age was assessed using a Shapiro-Wilk test and as it proved non-normally right-hand asymmetrically distributed (*p* < 0.001; coefficient of skewness of 1.7), it was reported with a median and interquartile range (IQR) and compared between groups using a Mann-Whitney U test. Ninety-five percent confidence intervals (CI 95%) for proportions were calculated using the Wilson score method [17]. A Pearson chi-square test and the associated version for a linear trend [18] were used for comparing prevalence of ocular diseases in the three age classes. All statistical hypotheses were two-tailed and significance level (α) was set at 0.05. Statistical analyses were performed in Statistica 12 (StatSoft Inc.).

## Results

The study population consisted of 425 females (69.1%) and 190 males (30.9%) ranging in age from 6 months to 33 years. The median age of males was significantly younger than that of females (median 2 years, IQR 2 to 4 years vs. median 5 years, IQR 2 to 8 years; *p* < 0.001). There were 348 young horses (58.9% female), 209 adult horses (87.0% female) and 58 elderly horses (67.2% female).

**Fig. 1:**
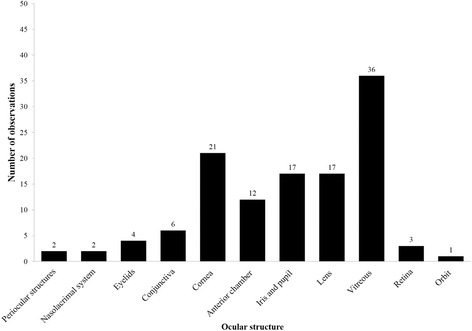
Prevalence of the lesions in eye structures in Arabian horses in breeding farms in Poland

**Fig. 2:**
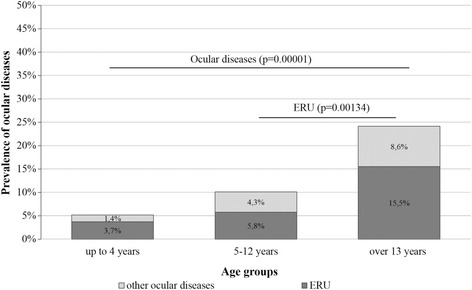
Prevalence of ERU and non ERU diseases in Arabian horses in breeding farms in Poland

A total number of 1228 eyes in 615 horses were examined. Two horses had one eye enucleated previously, one because of trauma and the other due to glaucoma and chronic pain. Abnormalities were found in 60 horses (9.8%; CI 95%: 7.7 to 12.4%) (Fig. 1.) with prevalence increasing with age: 6.0% (CI 95%: 4.0% to 9.0%) in young horses, 11.5% (CI 95%: 7.9% to 16.6) in adults and 25.9% (CI 95%: 16.3% to 38.4%) in elderly horses (*p* < 0.001) (Fig. 2.). The prevalence was uniform in all three breeding farms (*p* = 0.303), as well as in mares versus stallions (*p* = 0.103). In most examined horses with ocular disease, more than one pathology was noted. Therefore, the data is presented in figures and tables with reference to anatomical structures of the eye rather than to horses. The prevalence of observed symptoms is shown in Fig. 1, with the number of specific pathological changes in Table 1. Four horses presented with phthisis bulbi, one microphthalmia and one buphthalmia. Periocular structure deformations were detected in two horses: posttraumatic orbital fracture (one horse) and osteophyte formation at the base of the lacrimal bone (one horse). Nasolacrimal system pathologies were also diagnosed, including nasolacrimal duct obstruction and, rarely, lack of distal nasal lacrimal punctum due to imperforate nasal punctum. Other detected anomalies included: sarcoids affecting the upper eyelid in two horses, unilateral third eyelid paralysis, and in one horse, a scar resulting from a lower eyelid laceration. Conjunctivitis was diagnosed in six cases, all secondary to other coexistent pathologies present on the day of examination. The most common pathologies affecting the anterior globe were corneal scarring and ulcers (potentially indicative of previous keratitis) in 21 horses.

**Table 1:** Prevalance of pathological changes observed during ophthalmological examination

Pathological changes (some of them coexisting in one horse)	Number of findings
Phtisis bulbi	1
Microophthalmia	1
Buphthalmia	
glaucoma	1
Structure deformations	2
Conjunctivitis	6
Eyelid	
laceration	1
sarcoid	2
third eyelid paralisis	1
Nasolacrimal duct	
obstruction	1
lack of distal punctum	1
Corneal changes	
opacity	9
scar	17
ulcer	4
Anterior chamber	
hyphema	12
fibrin clots	17
Posterior synechiae	24
Iris	
atrophy	1
cysts	2
Lens	
luxation	1
mature cataract	4
immature cataract	15
Vitreal opacities	36
Optic nerve	
papilloedema	1
Retinal detachment	3

Four older horses had senile mature cataracts and 15 horses had immature cataracts. Opacities of the lens capsule (3 anterior lens capsule, 1 posterior) or lens cortex (10 horses with perinuclear and 3 with nuclear) were present in 17 cases.

One horse was diagnosed with hyphema and anterior lens luxation. Furthermore, 12 horses had hyphema and fibrin clots in the anterior chamber, 24 horses had anterior and posterior synechiae, 2 horses had iris cysts, and iris atrophy was observed in 1 horse. The most common abnormalities in the posterior segment of eyeball were vitreal opacities (36 cases) and subretinal/retinal haemorrhage with detached retina (3 cases). During fundus examination in one horse, papilloedema was observed.

Seven horses had non-visual eyes; one was completely blind because of bilateral mature cataracts, retinal detachment and vitreal opacities, and the remaining 6 had unilateral ocular abnormalities including mature cataracts, retinal detachment, phthisis bulbi, and in one case microphthalmia. In 36 horses pathologically vitreous changes were detected. In some older horses this was due to senile degeneration, but in general, changes were primarily caused by inflammation.

Thirty four horses demonstrated clinical signs characteristic for ERU: blepharospasm, corneal edema, aqueous flare, hypopyon, miosis, vitreous haze, iris fibrosis and hyperpigmentation, posterior synechia, corpora nigra degeneration, miosis, cataract formation, vitreous degeneration and discoloration, and/or retinal degeneration coupled with the history of recurrent or persistent uveitis in one or both eyes [13, 17, 18]. Thus, the clinical diagnosis of ERU was confirmed in 34 out of 60 (56.7%; CI 95%: 44.1% to 68.4%) horses with ophthalmic lesions and was the most common ocular disease (Table 2.). This corresponds to 5.5% (CI 95%: 4.0 to 7.6%) of Polish Arabian horse population. The prevalence did not differ between the three breeding farms (*p* = 0.779), nor did it differ between mares and stallions (*p* = 0.606). Prevalence of ERU was significantly higher in older horses (15.5%; CI 95%: 8.4% to 26.9%) compared to those younger than 13 years (4.5%; CI 95%: 3.1% to 6.6%) (*p* = 0.002). The difference between young and adult horses (3.7% and 5.8%, respectively) was not statistically significant (*p* = 0.286).

**Table 2:** Prevalence of ocular diseases in Arabian horses in breeding farms in Poland

Ocular disease	Number (%) of horses affected	Confident interval 95%
ERU	34 (5.5%)	4.0% - 7.6%
Post-traumatic lesions	5 (0.8%)	0.4% - 1.9%
Not ERU related cataract	20 (3.3%)	1.9% - 4.7%
Mature cataract	4	
Inmature cataract	15	
Congenital cataract	1	
Glaucoma	1 (0.16%)	
Total	60 (9.8%)	7.7% - 12.4%

## Discussion

The main goal of the study was to investigate and raise awareness of the prevalence of ocular diseases in horses in Poland. Since it is a new area of study in this country, the authors chose a valuable and well-known breed, the Polish Arabian, as the target population.

Data collection for this study was a significant challenge, requiring collaboration with study farms and veterinarians to gather clinical and historical information, including archived data, for 615 horses. The lack of published studies for reference also posed some difficulty. Previous studies describing ocular findings in horse populations were conducted as disease surveys limited to specific group of horses such as working horses [5], army horses [6] and horses in riding clubs [19]. Breed-specific studies have been limited to Thoroughbred racehorses [20] and Rocky Mountain Horses [21].

Several limitations of this study have to be taken into consideration. The primary issue is the structure of the Arabian horse population in Poland. There are approx. 4000 individuals (including 598 breeding mares and 227 stallions) kept at several hundred farms. Farm size varies from a just a few to over 200 individuals. The three farms enrolled in this study were the only locations at which more than 20 horses were kept. This represents a non-probability (convenience) sampling method, however the authors believe this is a sound approach as it allowed inclusion of the largest number of horses (15% of the Polish Arabian population) with detailed medical histories. It is also considered likely that this sample set was genetically representative of the entire population as 44% (264 of 598) of breeding mares were kept on the three farms and over 90% of the remaining 334 breeding mares on other farms were born on one of these 3 farms. Moreover, the ocular diseases under investigation were non-contagious, meaning that the concentrated population distribution of the sample set was unlikely to bias the results if compared to the larger majority of horses kept in tiny clusters at smaller farms. Neither general ocular disease prevalence nor ERU prevalence differ between the three study farms, therefore it was also unlikely to differ between other stables based on management differences.

The prevalence of ocular lesions reported in this study (9.8% of the investigated population), as well as the increasing prevalence observed in older horses, is consistent with most similar studies [4–7, 19–23]. However, it is difficult to compare the results obtained in our study with literature data due to substantial differences in study design (detection of the disease, description of eye lesions, variations in horse population), methods used (detailed ophthalmological examination, inspection only, or ophthalmological examination with the analysis of medical history), distinct forms of classification of ophthalmic disorders (usually categorized based on the type or place of lesions) as well as data presentation and description (lesions vs. horse with disease). Furthermore, other factors including climate, environment, husbandry practices, social and economic background, and the occurrence of infectious diseases and vectors (e.g. flies) also may influence the results.

Some epidemiological reports have been designed as retrospective studies of a specific disease, such as glaucoma [24] or ulcerative keratitis [25]. These studies were performed in patients at equine clinics selected for one disease, which could be diagnosed and described in detail. However, this type of study design does not give insight into the prevalence of disease in the general horse population. Most horses in the current study were not exhibiting acute signs of disease at the time of examination and were observed in their everyday living situation versus a clinic environment, adding validity to the results.

Epidemiological studies on the non-hospitalized population reveal multiple disease stages, although very rarely the acute phase. Instead, ongoing chronic pathological processes or evidence of previous ophthalmological disorders are commonly observed [4–7, 19–21, 23]. Untreated disease may extend to other eye structures, leading to visual impairment, which is often not identified by the owner due to patient adaptation [5, 19]. Use of the horse and the owner’s knowledge is critical in this situation. In an Australian study, vision-threatening eye diseases surprisingly were detected in 7.5% of horses without any reported history of vision impairment [19]. This was greater than previously perceived, and highlighted the importance of ocular examination as a part of any routine physical examination of horses. This has important implications for human safety as well, as riding horses with unknown vision impairments pose a considerable risk for humans as well as themselves.

Other studies have reported general eye lesions in specific groups of horses, such as horses undergoing pre-purchase examination or riding school inspection [6], patients of equine veterinary clinics [7, 21], geriatric horses [5, 20], healthy neonatal foals [26] or race horses [19]. These studies were focused on lesion description, which can result from multiple diseases. Progressive or cumulative effects of several processes were potentially present and specific diseases were not precisely diagnosed. Moreover, they were based on general inspection versus a detailed ophthalmological examination. Such study design allows evaluation of the risk for vision loss and possible consequences [6, 19], but only provides limited information on disease prevalence. This study was designed to focus on proper examination and diagnosis of ocular diseases using a group of horses with common genetic background (Arabian breeding horses) across a wide age distribution. Moreover, the detailed medical records and consistent veterinary oversight of this study population allowed for selection of horses without other health issues. To the authors’ knowledge there was no similar database source utilized for other published studies.

Trauma, glaucoma, and cataracts are the primary reported eye abnormalities [4, 6, 7, 19, 22, 23]. However, the most common cause of blindness is equine recurrent uveitis (ERU) which seems to be the most important ophthalmological problem in horses worldwide [3, 4, 8–11, 27–29]. Consistent with this, the current study demonstrates the importance of ERU in Arabian horses in Poland. Prevalence of ERU has been reported as 0% to 25% and even 40% in endemic areas [3, 4, 6, 8–10, 12], although other studies indicate that 8–10% occurrence is typical for most countries [5, 7, 8, 10]. Generally, the prevalence of eye disease in horses with potential threat to vision has been reported as below 1% up to 15% [4, 6, 7, 19, 21], rarely higher than 20% [22, 23]. However, subclinical ophthalmic lesions detected during ocular examination, but not noticed by owners or during routine physical examination, has been reported in up to 60–90% of examined horses [5, 6, 19, 20].

A cross-sectional study conducted in Ethiopia observed eye abnormalities in 23% of horses. Over 50% of affected eyes had visual impairment and ocular discharge. Moreover, ocular pain was evident in approximately 30% of horses but only a small number were receiving treatment or had been presented to a veterinary clinic with an eye problem [22].

Age predisposition for ocular diseases is widely accepted. In geriatric horses ocular lesions are common (approx. 60–90%). Vitreous degeneration, cataracts, and senile retinopathy or retinal degeneration are described most often [5, 20, 30]. The current study confirmed the higher prevalence of eye abnormalities in older horses, however, it did not exceed 25%. Interestingly, one published study claims that vision problems are less important for geriatric animals as they are no longer used for work or breeding [5]. One mare from our study population was blind for ten years due to mature bilateral cataracts, retinal detachment, and vitreous opacities. However, she was still a successful broodmare, likely due to management accommodations provided by her caretakers.

In foals, congenital ocular abnormalities pose the most serious problem. A high prevalence of such lesions has been reported in Rocky Mountain Horses [31] but appear rather uncommon in Standardbred foals [14, 26]. Acquired ophthalmic abnormalities as an ocular hemorrhage are relatively frequent [14, 26]. All foals examined in our study were older than 6 months, therefore details in the neonatal foal population are not described.

Our data indicate that ERU is the most prevalent disease in the Polish Arabian population, identified in 5.5% of 615 individuals examined, which corresponds to over 50% of horses with ophthalmic lesions. Breed predisposition for this disease has been demonstrated primarily in Appaloosas, but also to a lesser extent in European Warmbloods, draught breeds, Standardbred trotters and color-dilute horses [9, 12, 28, 29, 32–35]. As a breed, Arabians represent a population of average risk for ERU development, which must be taken into consideration for comparison with data from mixed breed populations in Europe.

Variation in ERU has been reported previously, ranging from 0% to 25% and even 40% in endemic areas. Although most studies indicate that prevalence of 8–10% is typical for Europe, we could not identify the primary source of this information and only sparse current epidemiological data are available [3–12]. Most recent studies describe lower ERU prevalence, such as 0.2% (2 from 805) of horses in Brazil [7], 0.8% (6 from 901) of horses in Iran [4], and 0.9% of horses (6 from 693) in United Kingdom [6]. Only the studies from United Kingdom [5] and Germany [36] reported similar prevalence to that indicated by our study; 6% (5 from 83 geriatric horses) and 7.6% (78 from 1014 animals) respectively. Some epidemiological studies describe uveitis in horses but without any information about the type of disorder, e.g. in 24 of investigated 500 horses in India (4.8%), uveitis cases constituted 24% of horses with ophthalmologic disorders [23]. ERU may be considered in these cases but cannot be diagnosed based only on the detected lesions without proper documentation of medical history as lesions caused by episodes of ERU and other reasons cannot be differentiated.

Taking into account the specific diagnosis, ERU is the most or second most common (after ocular trauma) reported ophthalmologic disease. In one Iranian study, ERU was diagnosed in 15% of horses with eye abnormalities and only eye injuries with various sequelae were more commonly reported [4, 7, 22]. However, the high occurrence of ERU in Polish Arabian horses with eye abnormalities of 56.7% is surprising, particularly given the research was conducted in breeding farms rather than veterinary clinics.

Lack of age and gender predilections for ERU have been reported previously [37]. Our study confirmed this finding. We found no significant differences related to sex and environment (farms), however, the prevalence of ERU increased with age and disease was diagnosed most frequently in horses older than 12 years. This could be explained by the type of the study, as a cross-sectional study decreases the probability of finding a horse experiencing an acute phase of the disease. Diagnosis was based on detected lesions and historical data. Because of the recurrent and progressive nature of ERU, the lesions develop with age, reflecting multiple episodes. Occurrence of ERU is spontaneous and recurrence is unpredictable. The disease tends to increase in severity with repeated episodes resulting in cumulative damage to the eye which can then be detected during ophthalmoscopic examination [10–12, 27, 28, 37, 38].

Similar lesions of the cornea can be caused by trauma, inflammation, glaucoma or episodes of uveitis [39]. Infectious disorders of eye may also lead to identical lesions if not managed correctly [40]. Trauma was reported as the most important cause of ocular lesions in working horses (36% of cases) [4, 7, 22]. In this study we observed post-traumatic lesions (according to medical history gathered from farm vets) in 0.8% of horses. This is comparable with other studies [5, 6, 19], however lesions indicating a history of chronic keratitis caused by other factors and coexisting with other lesions were detected in 20 horses. In racehorses in Japan ulcerative keratitis caused by different factors was the most frequent corneal and ocular disease but the incidence rate was very low 0.015% [25]. In contrast, corneal edema, opacity and scarring were diagnosed in 97.5% of horses older than 15 years [5]. This indicates that data from questionnaires and medical history is crucial for proper recognition and diagnosis of any abnormalities.

In this study cataracts unrelated to ERU were diagnosed in 3.3% of investigated horses and congenital cataracts were diagnosed in only one case. In most cases cataracts were diagnosed in horses with concurrent ocular abnormalities and in older horses, consistent with previous studies. Cataracts have been reported to affect 5–7% of horses [6, 15, 41] but some studies reveal significantly different prevalence of less than 1% [4, 7, 23], approx. 20% [5, 19] and even 97% in geriatric horses [20].

The current study shows low prevalence of glaucoma in horses (a single case, 0.16%). Primary glaucoma in horses has been reported rarely, and is most often secondary to ERU. Age greater than 15 years is the main risk factor [15, 23, 24].

## Conclusions

Ocular diseases in Arabian horses in Poland represent a significant welfare and economic concern. Equine recurrent uveitis appears to be the most common, however its prevalence in this population is lower than has been previously reported in Europe and the United States. Various ocular pathologies were observed, including severe conditions, confirming the presence and describing the prevalence of this important clinical problem among the study population. There was no observed sex or farm predisposition but the prevalence increases with age.

## Additional file

Additional file 1: Raw data. (XLSX 9 kb)

## Abbreviations

CI: Confidence intervals
ERU: Equine recurrent uveitis
IQR: Interquartile range

## References

[1] Strumiłło A.: Al Jawad, Konie Janowa: Dom Wydawniczy Benkowski; 2007. http://www.benkowski.home.pl.

[2] Bielański W (2008). Historia i współczesne zagadnienia hodowli i użytkowania koni w Polsce.

[3] Gilger BC, Deeg C (2011). Equine ophthalmology.

[4] Bazargani TT, Moaddab SH, Raoofi A, Masoudifard M, Bahonar AR (2011). Study of the prevalence and type of ophthalmic diseases among different breeds of horses in Tehran riding clubs. International Journal of Veterinary Research.

[5] Chandler KJ, Billson FM, Mellor DJ (2003). Ophthalmic lesions in 83 geriatric horses and ponies. Vet Rec.

[6] Grundon RA (2008). A retrospective comparison of eye lesions in two populations of horses: horses at pre-purchase examination and at riding school inspections. BrAVO Proceedings.

[7] Reichman P, de Olivera Dearo AC, Rodrigues TC (2008). Occurence of ophthalmologic diseases in horses used for urban cart hauling in Landrina. Ciencia Rural.

[8] Deeg CA, Hauck SM, Amann B, Pompetzki D, Altmann F, Raith A, Schmalzl T, Stangassinger M, Ueffing M (2008). Equine recurrent uveitis - A spontaneous horse model of uveitis. Ophthalmic Res.

[9] Lowe RC (2010). Equine uveitis: a UK perspective. Equine Vet J Suppl.

[10] Spiess BM (2010). Equine recurrent uveitis: the European viewpoint. Equine Vet J Suppl.

[11] Witkowski L, Cywinska A, Paschalis-Trela K, Crisman M, Kita J (2016). Multiple etiologies of equine recurrent uveitis – a natural model for human autoimmune uveitis: a brief review. Comp Immunol Microbiol Infect Dis.

[12] Gilger BC, Deeg CA, Gilger BC (2011). Equine Recurrent Uveitis. Equine ophthalmology.

[13] Thrusfield MV. Veterinary Epidemiology 3rd edition: Wiley-Blackwell; 2007.

[14] Labelle AL, Hamor RE, Townsend WM, Mitchell MA, Zarfoss MK, Breaux CB, Thomasy SM, Hall T (2011). Ophthalmic lesions in neonatal foals evaluated for nonophthalmic disease at referral hospitals. Javma-Journal of the American Veterinary Medical Association.

[15] Matthews AG (2000). Lens opacities in the horse: a clinical classification. Vet Ophthalmol.

[16] Wilkie DA (2010). Equine glaucoma: state of the art. Equine Vet J Suppl.

[17] Altman D, Machin D, Bryant T, Gardner M: Statistics with Confidence: Confidence Intervals and Statistical Guidelines, 2nd edn: BMJ Books; 2000.

[18] Zar JH (2010). Biostatistical analysis.

[19] Hurn SD, Turner AG (2006). Ophthalmic examination findings of thoroughbred racehorses in Australia. Vet Ophthalmol.

[20] Ireland JL, Clegg PD, McGowan CM, McKane SA, Chandler KJ, Pinchbeck GL (2012). Disease prevalence in geriatric horses in the United Kingdom: veterinary clinical assessment of 200 cases. Equine Vet J.

[21] Mellor DJ, Love S, Walker R, Gettinby G, Reid SWJ (2001). Sentinel practice-based survey of the management and health of horses in northern Britain. Vet Rec.

[22] Scantlebury CE, Aklilu N, Reed K, Knottenbelt DC, Gebreab F, Pinchbeck GL (2013). Ocular disease in working horses in Ethiopia: a cross-sectional study. Vet Rec.

[23] Thangadurai R, Sharma S, Bali D, Rana BP, Mahajan V, Samanta I, Hazra S (2010). Prevalence of ocular disorders in an Indian population of horses. J Equine Vet Sci.

[24] Curto EM, Gemensky-Metzler AJ, Chandler HL, Wilkie DA (2014). Equine glaucoma: a histopathologic retrospective study (1999-2012). Vet Ophthalmol.

[25] Wada S, Hobo S, Niwa H (2010). Ulcerative keratitis in thoroughbred racehorses in Japan from 1997 to 2008. Vet Ophthalmol.

[26] Barsotti G, Sgorbini M, Marmorini P, Corazza M (2013). Ocular abnormalities in healthy Standardbred foals. Vet Ophthalmol.

[27] Curling A (2011). Equine recurrent uveitis: classification, etiology, and pathogenesis. Compend Contin Educ Vet.

[28] Gerding JC, Gilger BC. Prognosis and impact of Equine Recurrent Uveitis. Equine Vet J. 2015. doi:10.1111/evj.12451. 10.1111/evj.12451 25891653

[29] Gilger BC (2010). Equine recurrent uveitis: the viewpoint from the USA. Equine Vet J Suppl.

[30] Nell B, Walde I (2010). Posterior segment diseases. Equine Vet J Suppl.

[31] Ramsey DT, Ewart SL, Render JA, Cook CS, Latimer CA (1999). Congenital ocular abnormalities of Rocky Mountain horses. Vet Ophthalmol.

[32] Deeg CA, Marti E, Gaillard C, Kaspers B (2004). Equine recurrent uveitis is strongly associated with the MHC class 1 haplotype ELA-A9. Equine Vet J.

[33] Fritz KL, Kaese HJ, Valberg SJ, Hendrickson JA, Rendahl AK, Bellone RR, Dynes KM, Wagner ML, Lucio MA, Cuomo FM (2014). Genetic risk factors for insidious equine recurrent uveitis in appaloosa horses. Anim Genet.

[34] Kulbrock M, Lehner S, Metzger J, Ohnesorge B, Distl O (2013). A Genome-Wide Association Study Identifies Risk Loci to Equine Recurrent Uveitis in German Warmblood Horses. PLoS One.

[35] Dwyer AE, Crockett RS, Kalsow CM (1995). Association of Leptospiral Seroreactivity and Breed with Uveitis and blindness in horses - 372 cases (1986-1993). J Am Vet Med Assoc.

[36] Szemes PA, Gerhards H (2000). Study on the prevalence of equine recurrent uveitis in the cologne-Bonn area. Praktische Tierarzt.

[37] Deeg CA, Kaspers B, Gerhards H, Thurau SR, Wollanke B, Wildner G (2001). Immune responses to retinal autoantigens and peptides in equine recurrent uveitis. Invest Ophthalmol Vis Sci.

[38] Faber NA, Crawford M, LeFebvre RB, Buyukmihci NC, Madigan JE, Willits NH (2000). Detection of Leptospira spp. in the aqueous humor of horses with naturally acquired recurrent uveitis. J Clin Microbiol.

[39] Cutler TJ (2004). Corneal epithelial disease. Vet Clin North Am Equine Pract.

[40] Brooks DE (2004). Inflammatory stromal keratopathies: medical management of stromal keratomalacia, stromal abscesses, eosinophilic keratitis, and band keratopathy in the horse. Veterinary Clinics of North America-Equine Practice.

[41] Matthews AG (2004). The lens and cataracts. Veterinary Clinics of North America-Equine Practice.

